# The antenna transcriptome changes in mosquito *Anopheles sinensis*, pre- and post- blood meal

**DOI:** 10.1371/journal.pone.0181399

**Published:** 2017-07-17

**Authors:** Qian Chen, Di Pei, Jianyong Li, Chengyu Jing, Wenjian Wu, Yahui Man

**Affiliations:** 1 College of Science, National University of Defense Technology, Changsha, Hunan, China; 2 State Key Lab on NBC Protection for Civilian, Research Institute of Chemical Defense, Beijing, China; Universidade Federal do Rio de Janeiro, BRAZIL

## Abstract

Antenna is the main chemosensory organ in mosquitoes. Characterization of the transcriptional changes after blood meal, especially those related to chemoreception, may help to explain mosquito blood sucking behavior and to identify novel targets for mosquito control. *Anopheles sinensis* is an Asiatic mosquito species which transmits malaria and lymphatic filariasis. However, studies on chemosensory biology in female *An*. *sinensis* are quite lacking. Here we report a transcriptome analysis of *An*. *sinensis* female antennae pre- and post- blood meal. We created six *An*. *sinensis* antenna RNA-seq libraries, three from females without blood meal and three from females five hours after a blood meal. Illumina sequencing was conducted to analyze the transcriptome differences between the two groups. In total, the sequenced fragments created 21,643 genes, 1,828 of them were novel. 12,861 of these genes were considered to be expressed (FPKM >1.0) in at least one of the two groups, with 12,159 genes expressed in both groups. 548 genes were differentially expressed in the blood-fed group, with 331 genes up-regulated and 217 genes down-regulated. GO enrichment analysis of the differentially expressed genes suggested that there were no statistically over represented GO terms among down-regulated genes in blood-fed mosquitoes, while the enriched GO terms of the up-regulated genes occurred mainly in metabolic process. For the chemosensory gene families, a subtle distinction in the expression levels can be observed according to our statistical analysis. However, the firstly comprehensive identification of these chemosensory gene families in *An*. *sinensis* antennae will help to characterize the precise function of these proteins in odor recognition in mosquitoes. This study provides a first global view in the changes of transcript accumulation elicited by blood meal in *An*. *sinensis* female antennae.

## Introduction

Mosquitoes are generally considered one of the most harmful vectors of many diseases caused by viruses and parasites due to their blood-feeding behavior [1]. *Anopheles sinensis* is an Asiatic mosquito species which transmits some of the most prevalent human parasitic diseases, including malaria and lymphatic filariasis, affecting humans in Southeast Asia [2, 3]. In addition, the species is highly susceptible to *Plasmodium vivax* [4], and was found an increasing vectorial capacity in recent outbreak of malaria in China [5]. Traditional methods such as insecticide-treated nets and indoor residual spraying are facing mosquitoes’ developing resistance and changing behavior [6]. Since female mosquitoes need blood to complete their oogenesis, the close contact of mosquitoes with their hosts is a crucial step for blood feeding and diseases transmitting. Therefore, understanding and exploiting the proximate mechanisms of host location in mosquitoes may help to reduce their interaction with human hosts and prevent the transmission of infectious diseases.

Chemosensation is a critical sensory modality in mosquitoes to locate hosts. Antennae are the main olfactory organs that respond to volatile odors and transmit chemosensory signals [7, 8]. The major peripheral olfactory proteins involved in odor recognition in insects antennae are binding proteins (odorant binding proteins (OBPs) and chemosensory proteins (CSPs)), membrane-bound chemosensory receptors (odorant receptors (ORs), ionotropic receptors (IRs), gustatory receptors (GRs), and sensory neuron membrane proteins (SNMPs)) [9–14]. With the identification of these chemosensory gene families from many mosquito species, the correlation between gene expression and behavioral observation has been facilitated [15–17]. However, regulation of these chemosensory genes remains largely unknown. Moreover, the studies of female *An*. *sinensis* chemosensory biology are particularly lacking.

In the past few years, transcriptome sequencing of *Anopheles gambiae* has led to the abundance quantitation of chemoreceptor genes expressed in antennae of various mosquito species [18–21]. The publically available data sets created from these studies have provided significant insights into the molecular mechanisms of mosquito chemosensory driven behaviors. While several studies have examined transcript abundance in *An*. *sinensis* [22–24], none of them has focused on chemoreceptive tissues.

The present study mainly focuses on identifying genes related to host detection and location from *An*. *sinensis* antennae. As far, only a few odor-recognition-related genes from *An*. *sinensis* have been characterized [25, 26]. It is hoped that a comprehensive identification of host-location-related genes in *An*. *sinensis* genome will improve our understanding on the molecular mechanism of chemosensory driven behaviors. Manipulation with these genes may offer some effective approaches for malaria control. In this study, we have sequenced mRNA extracted from *An*. *sinensis* female antennae to compare the transcriptome profiles of non-blood-fed females with that of blood-fed females five hours after blood feeding. Our results help to refine the chemoseneory genes annotation of *An*. *sinensis*, improve our understanding of the molecular processes induced by a blood meal in antennae, and establish a data set that can be used for further researches.

## Methods

### Mosquito strains and rearing

The colony of *An*. *sinensis* used in this study was reared in the Chinese Center for Disease Control and Prevention (CDC, Beijing). Mosquitoes were maintained at 28±1°C and 70–80% relative humidity, with a 12:12 h. light/dark photoperiod. Larvae were fed a 1:3 mix of finely ground pork liver powder (self-made) and yeast powder. Adults were maintained with free access to a 10% sugar solution. Mosquitoes aged 4 days after eclosion were either allowed to take a blood meal for half an hour from anesthetized mice (the group B) or continued with sugar feeding (the group S). Five hours after blood feeding, 50 female antennae of each group were collected into TRIzol reagents (Invitrogen). The above steps were repeated twice until six samples were obtained from three biological replicates. The mice used in blood feeding were anesthetized by sodium pentobarbital anesthesia, the protocol was approved by the Committee on the Ethics of Animal Experiments of the National University of Defense Technology.

### RNA extraction and RNA sequencing

Three separate samples of the antennae from the each treatment group were processed for RNA extraction using the TRIzol reagent according to the manufacturer’s protocol. After quantity and quality checking, a total of 3 μg RNA per sample was enriched for the RNA sample preparations. A total of six sequencing libraries were generated respectively using the NEBNext^®^ Ultra^™^ RNA Library Prep Kit for Illumina^®^ (NEB, USA) according to the manufacturer’s instructions, the index codes were added to the attribute sequence for each sample, the Agilent Bioanalyzer 2100 system was used to evaluate the quality of libraries.

Sample clustering was performed on a cBot Cluster Generation System using the TruSeq PE Cluster Kit v3-cBot-HS (Illumina) according to the manufacturer’s instructions. After then, library preparations were sequenced on the Illumina Hiseq platform, yielding a paired-end reads of 125 bp/150 bp.

### Data processing and gene expression level quantification

The raw data in the fastq format (raw read) were processed by the internal perl scripts. In this step, the reads containing the adapter, poly-N, and with low quality were deleted, Q20, Q30 and GC content of the clean reads were calculated. All of the following analyzes were based on high quality clean reads. Raw data have been deposited at the Short Read Archive (NCBI) under accession number PRJNA377961.

The *An*. *sinensis* genome (version: AsinC2) [27] and genomic annotation files were downloaded from the web site of Vectorbase (www.vectorbase.org). The index file of the reference genome was checked by Bowtie v2.2.3 and the paired-end clean reads were aligned to the *An*. *sinensis* China strain genome by TopHat v2.0.12 [28]. The TopHat was chosen as a mapping tool because it can generate splicing database based on the existing gene model annotation file, it is better than other non-splicing mapping tools [29]. Known and new transcripts from the TopHat alignment were reconstructed and identified by the Cufflinks v2.1.1 Reference Annotation Based Transcript (RABT) assembly method [30]. HTSeq v0.6.1 was used to calculate the reads mapped to each gene [31], the number of fragments mapped for every thousand bases of gene length for every million fragments sequenced (FPKM) was used as an indicator to measure the gene expression levels. In this study, only genes with an value of FPKM >1 were considered to be expressed, and retained for expression comparisons [32].

### Differential transcript abundance calculation

Differential expression analysis was performed by the DESeq R package (1.18.0) for two groups (three biological replicates per group) [33]. DESeq provides statistical routines for quantitative measurement of gene expression based on the negative binomial distribution. The normalized read counts were calculated for each sample. The resulting P-values were adjusted by the Benjamini and Hochberg methods to control false discovery rate (FDR). An FDR-adjusted P-value (padj) cut-off of ≤ 0.05 was used to identify differentially expressed genes. In this study, the three repetitions of S group were considered the control group, compared with the control group, the difference levels showing in B sample were calculated with the log_2_ (fold change), the value of which > 0 means expression up-regulated, and vice versa [34].

### RNA-seq data validation by real-time quantitative RT-PCR

A total of 10 differential expression genes between S and B groups were chosen randomly for real-time quantitative PCR analysis. Total antenna RNA was extracted using TRIzol from females which were treated the same as those for RNA-seq samples (S and B). After DNAse I (Invitrogen) treatment, cDNA synthesis were performed by the Transcriptor First Strand cDNA Synthesis kits (Roche, Germany). Real-time quantitative PCR was performed on LightCycler 480 (Roche, Germany) using Light Cycler 480 SYBR Green I Master (Roche, Germany) according to the manufacturer’s instruction. The experiments were conducted with three biological replicates, each in triplicates. Fold-changes in gene expression level between S and B were analyzed using the 2^-ΔΔCT^ method, The ribosomal protein S7 (rps7) was used as the endogenous control according to previous studies [35]. The results were calculated directly by LightCycler 480 SW 1.5 software. Student’s t-test was used to compare the expression values of blood-fed sample and non-blood-fed sample, and p<0.05 indicated significant difference. For qPCR analysis, gene-specific primers were designed using the Primer Premier 5.0 and are listed in S1 Table.

### Gene ontology (GO) enrichment

Annotation information and gene ontology (GO) data were obtained from the database of gene ontology (www.geneontology.org). GO enrichment of different sets of differentially expressed genes was performed using the GOseq R package, so that the gene length bias could be corrected. GO terms with a corrected P-value less than 0.05 were considered to be significantly enriched by the differentially expressed genes [36].

### Olfactory related genes identification and phylogenetic analysis

The gene sequences of all the *Anopheline* species and *Drosophila melanogaster* in PFAM protein family database (http://pfam.xfam.org) based on the pfam ID of OBP (PF01395), CSP (PF03392), OR (PF02949), IR (PF00060), GR (PF08395) and SNMP (PF01130) were respectively downloaded and were used as query to search the genome of *An*. *sinensis* China strain (AsinC2) on the website of vectorbase with the hmmer tool [37]. The putative chemosensory genes were then used to analyze read counts, and only genes with FPKM>1 from our sequence alignment data were used to analyze differential expression. Neighbor-Joining trees based on DNA alignments were constructed for each of OR/GR/IR/OBP/CSP families using the MEGA 5.0 program, respectively, with 1,000 bootstrap replicates.

## Results

### Basic sequencing data

**Fig 1:**
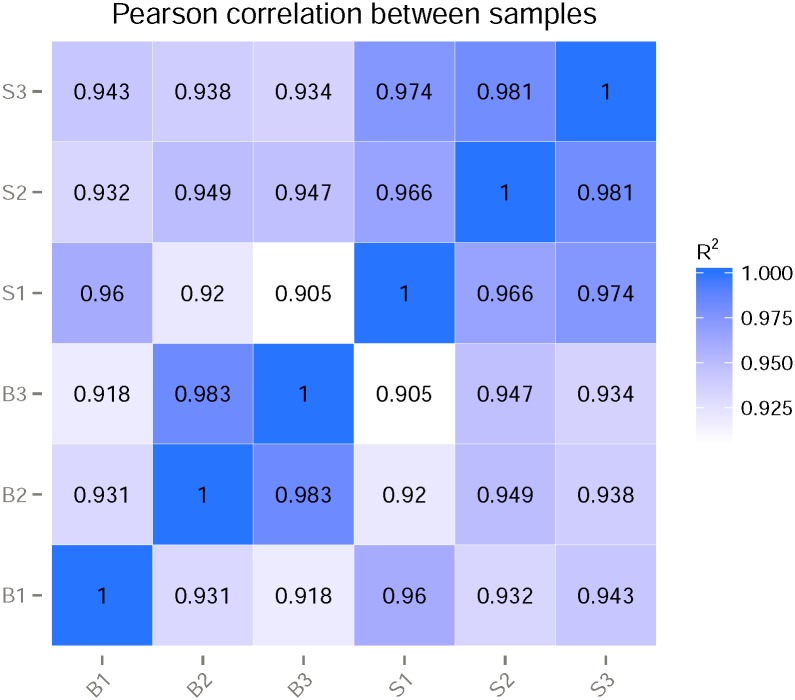
Correlation test among the biological repetitions. B1, B2, B3: three blood-fed replicate libraries. S1, S2, S3: three sugar-fed replicate libraries. R^2^: the square of Pearson correlation coefficient.

Six RNA-seq libraries were prepared with the total RNA exacted from S and B samples and were sequenced on Illumina Hiseq platform. A total of more than 292 million raw reads were generated from the six samples. After filtering, about 281 million clean reads were obtained, and the counts of these reads ranged from about 41 to 51 million for each sample. For each sample, about 30 million reads (approximately 70%) could be mapped to the *An*. *sinensis* genome (version:AsinC2) (Table 1). This indicates that a large number of transcripts have not been annotated. Therefore, our data were used not only for measuring antenna differentially expressed chemosensory genes, but also for identifying novel transcripts. Both known and novel genes from TopHat alignment were constructed using Cufflinks v2.1.1 Reference Annotation Based Transcript (RABT) assembly method. The alternative splicing events were found by the software of Asprofile v1.0. This approach increased 1828 transcripts in antennae (S2 Table). In total, the sequenced fragments created 21,643 genes, 1,828 of them are novel (S2 Table). 12,861 of these genes were detected in one or both samples (FPKM >1.0) (S2 Table). The Pearson correlation coefficients was used to confirm the correlation between the three biological repetitions in each condition (Fig 1), in most case, the square of the Pearson correlation coefficients (R^2^) was larger than 0.92 except the pair of B1 and B3 (R^2^ = 0.918). Therefore, the correlation from parallel libraries were considered reasonable for combining to further analyses.

**Table 1:** Overview of *An*. *sinensis* transcriptome and mapped reads.

Condition^1^	Replicate	Raw reads	Clean reads	Total mapped^2^	Multiple mapped^3^	Uniquely mapped^4^
B	B1	52763740	51090768	36903327(72.23%)	1250481 (2.45%)	35652846 (69.78%)
	B2	44548754	41823354	28899703(69.1%)	974823 (2.33%)	27924880 (66.77%)
	B3	48835826	46111894	31780383(68.92%)	993716 (2.16%)	30786667 (66.77%)
S	S1	51108400	49423120	35302157(71.43%)	1399956 (2.83%)	33902201 (68.6%)
	S2	46040234	44688370	31687674(70.91%)	1092732 (2.45%)	30594942 (68.46%)
	S3	49539572	47936824	34642577(72.27%)	1169409 (2.44%)	33473168 (69.83%)

^1^ Blood-fed (B) or sugar-fed (S).

^2^ Total number and percentage of reads mapped to reference genome.

^3^ Reads mapping to multiple position on the *An*. *sinensis* genome.

^4^ Reads mapping to a unique position on the *An*. *sinensis* genome.

### Differential transcript accumulation between non-blood-fed and blood-fed *An*. *sinensis* females

To examine the significance of difference between two groups, we analyzed the data by DESeq package, which not only correct the error cause by read length, but also normalize the data to give mean read counts of transcripts [38]. Thus, we were able to evaluate differential levels of the expressed genes in a more biologically meaningful way.

**Fig 2:**
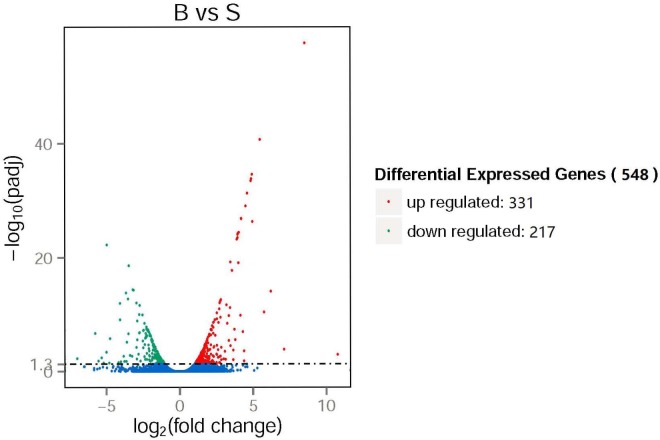
Differential expressed genes in *An*. *sinensis* female antennae. Volcano plot showing the relative expression levels of genes in sugar-fed (S) and blood-fed (B) female antennae. The x-axis represents the log_2_(fold change) for each gene of the *An*. *sinensis* antennae transcriptome. The y-axis represents the negative log10 of the FDR-adjusted P-value. Red dots (n = 331) represent genes up-regulated in B group with statistical significance. Green dots (n = 217) represent genes down-regulated in B group. Blue dots represent genes that fell outside one or both of these significance criteria.

**Fig 3:**
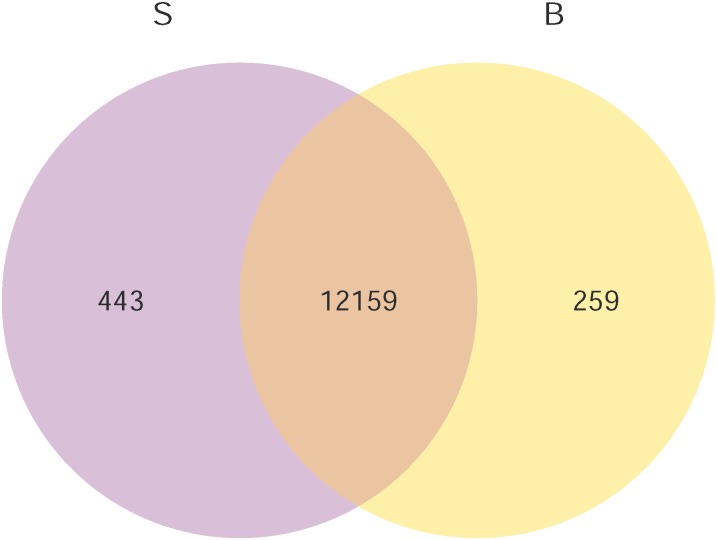
Gene expression pattern comparison of S and B sample in *An*.*sinensis*. Proportional Venn diagrams showing the comparisons made in this study. Overlap represents subset of genes that are expressed in both samples.

We calculated the value of padj ≤ 0.05 to identify differentially expressed genes, moderated log_2_ (fold change) to characterize the differential levels of these identified genes. These analyses showed that a total of 548 genes within the 12,861 expressed genes were identified as differential expressed genes between S and B groups. A volcano plot representing highly expressed genes respective in the S and B groups is shown in Fig 2, the detail gene list is shown in S3 Table. There were 331 and 217 genes were up- or down- regulated, respectively, following a blood meal. Detailed examination of the 331 up-regulated genes revealed that 5 were up-regulated more than 50-fold in B group, while the majority (261 genes) showed less than a 5-fold increase. Among the genes showing down-regulation following a blood meal, 172 of them were reduced from 2- to 5-fold. Only 2 genes were decreased more than 50-fold. For the ten differential expressed genes selected randomly, the results of qRT-PCR conducted on them were confirmed that both the direction and the magnitude of changes between S and B groups were agree with the RNA-seq analysis (Table 2). We also identified genes showing overlapping or specific expression in the two groups, 259 transcripts were expressed exclusively in B, while 443 were detected exclusively in S. 12159 genes were expressed in both groups. The venn diagram was displayed in Fig 3.

**Table 2:** qRT-PCR validation of RNA-seq data on a random selection of ten genes.

Vectorbase ID	RNA-seq			qPCR		
	Corrected readcounts^1^		log_2_(fold change)^2^	Normalized expression value^3^		log2(fold change)^4^
	B	S		B (std)	S (std)	
ASIC012251	1201.36	7896.29	-2.72	1.97 (0.24)	4.59 (1.74)	-1.22*
Novel00101	16.68	50.72	-1.60	0.54 (0.02)	2.22(1.36)	-2.03*
ASIC003215	210.51	535.48	-1.35	1.67×10^−2^ (3.51×10^−3^)	6.60×10^−2^ (3.95×10^−2^)	-1.98
ASIC014160	1080.60	2417.06	-1.16	1.26×10^−3^ (0.98×10^−3^)	4.31×10^−3^ (2.11×10^−3^)	-1.77*
ASIC008765	1505.19	703.64	1.10	3.57 (0.39)	1.89 (0.53)	0.92*
ASIC021000	1671.00	751.81	1.15	3.83×10^−2^ (1.22×10^−2^)	2.56×10^−2^ (0.34×10^−2^)	0.58*
ASIC021477	463.68	197.63	1.23	5.72×10^−3^ (4.51×10^−3^)	3.99×10^−3^ (2.64×10^−3^)	0.52
Novel00619	844.47	346.24	1.29	3.97 (1.09)	0.94 (0.63)	2.08*
ASIC006964	43498.82	9650.45	2.17	8.02×10^−2^ (5.39×10^−2^)	3.35×10^−2^ (1.58×10^−2^)	1.26*
ASIC007449	926.35	6.61	7.13	1.30 (0.67)	2.12×10^−2^ (0.74×10^−2^)	5.94*

^1^ The corrected number of total reads (normalized by DESeq) identified in libraries prepared from B and S samples.

^2^ log_2_(fold change): log_2_(group B/group S)

^3^ Normalized expression value as 2^ΔCT^, with the expression of the rps7 gene used to normalize values. Normalized expression values for B and S groups were obtained as an average (±standard deviation) over three biological samples.

^4^ Average fold changes as log_2_ (normalized expression value B/normalized expression value S). The significance of the difference in expression value between B and S mosquitoes was calculated using the t-test.

*P <0.05.

### Gene ontology (GO) enrichment

In order to investigate the major function categories represented in olfactory-related genes identified above, GO enrichment analysis was performed. Overall, we found that there were no statistically over represented GO terms among down-regulated genes in blood-fed mosquitoes.

**Fig 4:**
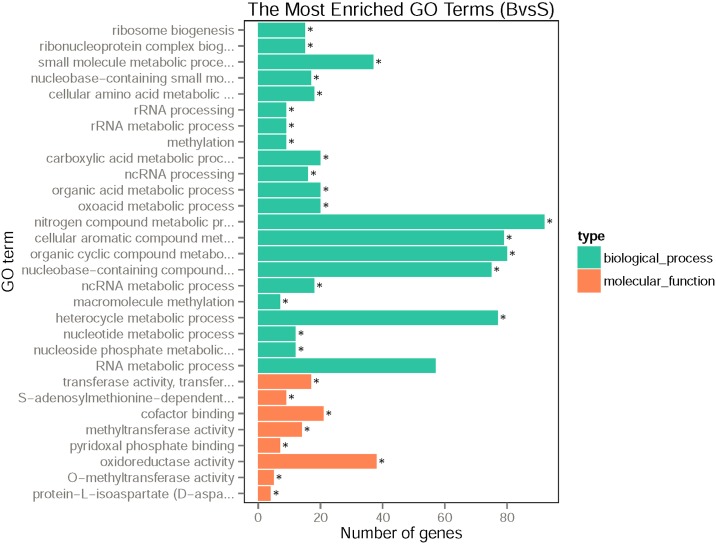
Gene ontology (GO) term enrichment of up-regulated genes in B group. x-axis represent the number of up-regulated genes in B group, y-axis represent the GO terms; Green bars represent biological process, Orange bars represent molecular function; “*” represent the significantly enriched terms (corrected P-value less than 0.05).

The functional enrichment analysis identified 29 statistically enriched GO terms among the 331 up-regulated genes in blood-fed mosquitoes (Fig 4), 21 of them belong to the biological process categories. Among these 21 enriched GO terms, nitrogen compound metabolic process (GO:0006807), organic cyclic compound metabolic process (GO:1901360), cellular aromatic compound metabolic process (GO:0006725), heterocycle metabolic process (CO:0046483) and nucleobase-containing compound metabolic process (GO:0006139) were the predominant GO terms, with 92, 80, 79, 77 and 75 transcripts, respectively. The results suggest that many of the up-regulated genes are involved in several metabolic processes. The remaining 8 statistically enriched GO terms associated with the various molecular functions, among them oxidoreductase activity (GO:0016491) and cofactor binding (GO:0048037) were most enriched, with hits of 37 and 21.

### Olfactory-related antennal transcripts

**Fig 5:**
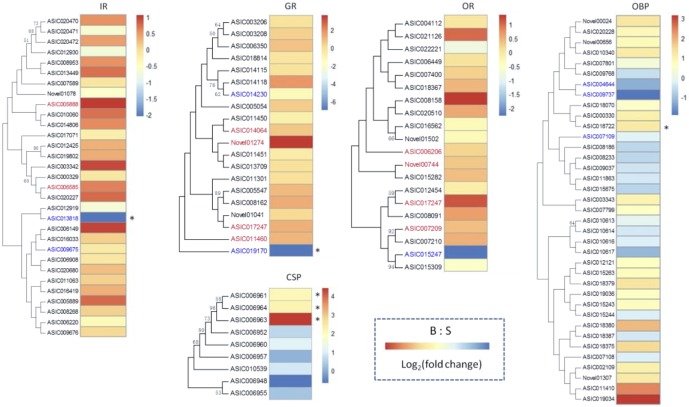
Differential antennal abundances of chemosensory genes. Heat maps for the five most abundant chemoreceptor gene families in the antenna. Only transcripts that were detectable in one or both species are displayed. Colors indicate a normalized log_2_(fold change), denoting enrichment in either S group (blue) or B group (red); Chemoreceptors are color coded if they were only classified as being detectable in S (blue) or in B (red). “*” marks the genes of significant differential abundance with the FDR-adjusted P-value (padj) less than 0.05.

Previous studies suggest that blood feeding alters sensitivity of olfactory receptor neurons in mosquitoes [39, 40]. Modulation of odor sensitivity could result from changes in expression of chemosensory genes. Thus, we compared the antennal transcriptome profiles of S and B groups for chemosensory genes in each of the OBP, CSP, OR, IR, GR and SNMP families to identify potential differential expression (Fig 5).

OBPs and CSPs are involved in the solubilization and transport of hydrophobic odorant. OBPs were the most abundant among these genes. We identified 55 OBPs genes from the *An*. *sinensis* genome (version: AsinC2) on the website of vectorbase and 3 novel transcripts from our sequencing, 37 of these 58 genes were identified to be expressed in the antenna (FPKM >1) (S4 Table). 3 transcripts (ASIC004644, ASIC009737 and ASIC007109) were detected exclusively in non-blood fed mosquito antenna. In the differential expression analysis, one member of the 37 expressed OBP family (ASIC018722) exhibited up-regulation in blood-fed mosquito with the padj<0.05. We identified 9 CSP genes from the *An*. *sinensis* genome, all of them were expressed in antenna. 30% (ASIC006961, ASIC006963 and ASIC006964) of them exhibited up-regulation in blood fed mosquito.

We identified 64 GRs from *An*. *sinensis* genome and 2 novel transcripts from our sequencing. 20 of these 66 genes were expressed in antenna, 2 (ASIC014230 and ASIC019170) were expressed exclusively in non-blood fed mosquito, and 4 (ASIC014064, Novel01274, ASIC017247 and ASIC011460) were expressed only in B sample. For IRs, 72 genes were identified from the genome of *An*. *sinensis*, and 2 novel transcripts from our sequencing. 31 of these 74 genes were detected in one or both samples, in which, 2 IRs (ASIC013818 and ASIC009675) were expressed exclusively in non-blood fed mosquito, and 2 (ASIC005888 and ASIC006585) were expressed only in B sample. Only one member each of GR (ASIC019170) and IR (ASIC013818) family displayed down-regulated in blood fed mosquito respectively.

OR is the best characterized chemosensory gene family in insects. Many functional researches on ORs have been conducted in both *D*. *melanogaster* and *An*. *gambiae*. In our analysis, 55 ORs were found from the genome on the website of vectorbase, 2 novel transcripts were identified in our sequencing. In addition, one of these 57 genes were detected exclusively in S sample (ASIC015247), while 4 of them were expressed in B sample (ASIC006206, ASIC017247, ASIC007209 and Novel00744). No OR genes showed statistically different differential expression.

Insects SNMPs, which are suggested to play a significant role in chemoreception, are homologs of the vertebrate CD36 transmembrane protein family. There are two SNMP subfamilies in insect: SNMP1 and SNMP2. In this study, we only identified SNMP2 from *An*. *sinensis* genome and antenna (ASIC016308). In the differential expression analysis, it cannot be found statistically significant differential expression (see S4 Table, not shown in Fig 5), with a padj = 0.93174.

In order to reveal the relationship between the expression pattern and evolution, phylogenetic tree among the chemosensory genes were built to compare with the expression differences of each receptor. However, there were no obvious relationships can be draw. Future studies with improved integrity of *An*. *sinensis* genome and perhaps identification of new chemosensory genes may provide more complete view of chemosensory gene expression in antenna pre- and post-blood meal.

## Discussion

So far, only a handful of studies describing the transcriptome of *An*. *sinensis* have been reported. One study has utilized 454 GS FLX system to analyze the samples from different developmental stages in *An*. *sinensis* and examine the transcriptome profiles between the mosquitoes resistance and susceptible to deltamethrin [23]. To provide a comprehensive and complete transcriptome of *An*. *sinensis*, a de novo transcriptome sequencing (illumina) investigation of eggs, larvae, pupae, male adults and female adults RNA, has been performed [22]. A transcriptome analysis on *An*. *sinensis* was performed earlier by large-scale EST sequencing to analyze the whole-body gene expression profiles of females treated with the apoptosis-inducing agent actinomycin D [24]. In this study, we examined the expression profiles of the peripheral chemoshensory tissues of *An*. *sinensis* to identify early changes before and five hours after blood feeding.

Antenna is an extremely important olfactory related organ, in which many specific cellular signaling events are involved with female mosquito host seeking. We are interested in exploring the molecular components that modulate the sensitivity to host odor in antenna of females, inasmuch as a pattern of small changes in the expression levels of multiple chemosensory genes may combinatorially shift the odor sensitivities in *An*. *gambiae* antenna [41]. We performed a comprehensive analysis comparing the transcriptomes of non-blood-fed (S) and blood-fed (B) female *An*. *Sinensis*, and identified several novel chemosensory protein coding genes.

With respect to OBPs, a small soluble semiochemical binding proteins, our analysis identified 58 OBP genes (55 from the known genome and 3 novel from our sequencing), with 37 being expressed in the antenna. The number of OBPs is lower than previously reported (64 putative OBP genes) at the whole genome level of *An*. *sinensis* [25]. A reason for the difference between these two studies may be the using of different reference genome: self-constructed genome in the previous study vs. vectorbase (AsinC2) in this study. An alternative explanation would be that some of these 64 OBP-coding genes previously reported in the genome of *An*. *sinensis* are not expressed in antennae but in other tissues. In addition to OBPs, we analyzed another family of odorant transporters, the CSPs, and their expression pattern, in *An*. *sinensis* antenna for the first time. There’s almost no previous study on GRs and IRs in *An*. *sinensis* except an interspecific analyses of the chemoreceptor superfamilies from the 18 species of *Anopheline* mosquitoes [26]. The present study identified IR and GR genes from genome of *An*. *sinensis* (version: AsinC) and by antenna transcriptome sequencing, and further compared their expression pattern in response to blood feeding. For the principal chemosensory gene classes, the relative levels of OR genes were invariant between S and B groups. The subtle distinctions in the expression are consistent with what reported in previous studies [41]. However, our RNA expression data revealed that the variety of the chemoreceptor (OR, IR and GR) genes was not conserved in these two groups, although the non-conservation could stem from that the FPKM>1 was used as gene expression threshold in our study. The non-conservative proteins expressed exclusively in the two conditions and the differential expressed proteins after blood feeding may be involved in host detection and/or host seeking. The certain role of these identified genes in host seeking in mosquitoes need to be further characterized by more functional and behavioral assays.

As far as we know, this is the first comprehensive identification and comparative research on the chemosensory gene repertoire between pre- and post-blood feeding in *An*. *sinensis*. Antenna is a key organ in mosquito behaviors, it serve as a prime target for mosquito control. Exploring its physiological function may provide a novel vector control strategies. In future, we will extend our study on the *An*. *sinensis* antenna transcriptome by increased sequencing methods combining with behaviors researches under different physiological conditions.

## Supporting information

S1 Table List of primers used for quantitative real-time RT-PCR analysis. (XLSX)

S2 Table Transcripts identified in *An*. *sinensis* female antennas in our sequencing. (XLSX)

S3 Table Differential expressed gene sets between non-blood fed (S) and blood fed (B) *An*. *sinensis* females. (XLSX)

S4 Table List of the expressed olfactory-related genes (OBP/OR/IR/GR/CSP/SNMP) in one or both samples (S and B), and their statistical comparisons in expression differences. (XLSX)
